# Does Baseline Diuretics Use Affect Prognosis in Patients With COVID-19?

**DOI:** 10.7759/cureus.15573

**Published:** 2021-06-10

**Authors:** Nirmal Guragai, Rahul Vasudev, Kevin Hosein, Habib Habib, Biren Patel, Parminder Kaur, Bhavik Patel, Melvin Santana, Sherif Elkattawy, Muhammad Atif Masood Noori, Islam Younes, Ramez Alyacoub, Balraj Singh, Raja Pullatt, Preet Randhawa, Fayez Shamoon

**Affiliations:** 1 Cardiology, St Joseph University Medical Center, Paterson, USA; 2 Internal Medicine, St Michael’s Medical Center, Newark, USA; 3 Internal Medicine, Rutgers-New Jersey Medical School/Trinitas Regional Medical Center, Elizabeth, USA; 4 Internal Medicine, Rutgers-New Jersey Medical School/Trinitas Regional Medical, Elizabeth, USA; 5 Internal Medicine, Dow Medical College, Karachi, PAK; 6 Hematology/Oncology, St Joseph's University Medical Center, Paterson, USA; 7 Cardiology, Trinitas Regional Medical Center, Elizabeth, USA; 8 Cardiology, St Michael’s Medical Center, Newark, USA

**Keywords:** coronavirus disease 2019 (covid-19), diuretics, ace inhibitors and angiotensin receptor blockers, anticoagulation, mortality, severity

## Abstract

The rapid emergence of coronavirus disease 2019 (COVID-19) has become the biggest healthcare crisis of the last century, resulting in thousands of deaths worldwide. There have been studies that evaluated the role of angiotensin-converting enzyme (ACE) inhibitors (ACEi) and angiotensin receptor blockers (ARBs) in treating patients with COVID-19. However, the prior use of diuretics and their effect on mortality in this setting remains unknown. The aim of the study was to evaluate the effect of diuretics in patients admitted with COVID-19. The current study was conducted between March 15, 2020, and April 30, 2020, during the COVID-19 pandemic in three different hospitals in Northern New Jersey, USA. The primary outcome was survival or in-hospital mortality from COVID-19 from the day of admission. The secondary outcome was severe or non-severe illness from COVID-19. This retrospective study included a total of 313 patients with a median age of 61.3 ± 14.6 years. There was a total of 68 patients taking diuretics at home and 245 patients who were not taking diuretics. There was a total of 39 (57.35%) deaths in patients taking diuretics as compared to 93 (37.96%) deaths in patients not taking diuretics (p-value 0.0042). Also, 54 (79.41%) patients who took diuretics had severe COVID-19 illness as compared to 116 (47.35%) who did not take diuretics (p-value <.0001). However, after adjusting for the confounding factors, there was no difference in mortality or severity of illness in COVID-19 patients taking diuretics at the time of admission. In conclusion, there was no effect of the baseline use of diuretics in the prognosis of COVID-19.

## Introduction

The rapid emergence of coronavirus disease 2019 (COVID-19) from Wuhan City, Hubei Province, China, has become the largest healthcare crisis of the last century, resulting in thousands of deaths worldwide [1]. The clinical spectrum of severe acute respiratory syndrome coronavirus 2 (SARS-CoV-2) infection appears to be wide, encompassing asymptomatic infection, mild upper respiratory tract illness, and severe viral pneumonia with respiratory failure and even death.

Several risk factors associated with severe COVID-19 have been identified, including older age, male sex, presence of comorbidities, low oxygen saturation, and abnormal lab findings [2-3]. Many infected patients, however, present with mild symptoms and recover quickly. Some studies have evaluated the role of angiotensin-converting enzyme (ACE) inhibitors (ACEi) and angiotensin receptor blockers (ARBs) and prior anticoagulation use in the treatment of these patients [4-5]. However, prior use of diuretics and their effect on mortality in COVID-19 remains unknown. The aim of the study was to evaluate the effect of baseline diuretics use in patients admitted with COVID-19.

## Materials and methods

The study was conducted between March 15, 2020, and April 30, 2020, during the COVID-19 pandemic in three different hospitals in Northern New Jersey, USA. The study complied with the edicts of the Declaration of Helsinki and was approved by the Ethics Committee of the respective institutions. In this retrospective, multicenter study, COVID-19 was confirmed with a reverse transcriptase-polymerase chain reaction (PCR) assay performed on nasopharyngeal swab specimens. Historical and laboratory data were manually abstracted from the electronic health records of the three different hospitals and were carefully reviewed and analyzed by trained physicians. Data collection included patient demographic information, baseline comorbidities, prior use of anticoagulation and diuretics, smoking status, initial QTc on electrocardiogram, initial lab, including serum potassium, magnesium, peak troponin, brain natriuretic peptide (BNP), liver enzymes, need for intensive care unit (ICU) admission, mechanical ventilation, and length of stay. The comorbidities included hypertension (HTN), diabetes mellitus, coronary artery disease (CAD), congestive heart failure (CHF), chronic obstructive pulmonary disease, asthma, chronic kidney disease (CKD), cancer, and immunosuppression. Patients who were taking diuretics, including prior to the admission, were included in the diuretics group. All data were cross-checked. Missing and uncertain records were excluded if they could not be provided or clarified by the involved healthcare providers and their families.

We categorized COVID-19 patients into two groups: a) severe and b) non-severe based upon the acuity of presentation. Severe COVID-19 was defined as septic shock or severe pneumonia and/or acute respiratory distress syndrome requiring ICU admission. Severe pneumonia was defined as pneumonia that causes systemic signs and requires noninvasive or invasive ventilation. A decision to intubate or transfer to ICU was at the discretion of the attending physician. The primary objective of this study is to determine the effect of baseline use of diuretics on mortality in hospitalized COVID-19 patients. The secondary objective is the effect of baseline use of diuretics on the severity of illness, based upon the above, prespecified criteria in hospitalized COVID-19 patients.

We utilized SAS 9.4 (SAS Institute Inc, Cary, NC) for all the statistical analyses. Data were expressed as counts and percentages for categorical variables, and Pearson’s chi-squared test was used to test differences between the patients who took or did not take diuretics. Normally distributed continuous variables were expressed with mean ± standard deviation and the student’s T-test was used to test the difference between these two groups. Non-normally distributed continuous variables are presented with median and interquartile range (IQR) and the Wilcoxon rank-sum test was used. Statistical significance was defined if the null hypothesis could be rejected at P < 0.05. A multivariate logistic regression analysis was performed to adjust for variation in baseline characteristics. In this model, we adjusted for age, gender, blood urea nitrogen (BUN), coronary artery disease (CAD), congestive heart failure (CHF), cerebrovascular accident (CVA), and chronic kidney disease (CKD).

## Results

In this retrospective, three-center study between March 15, 2020, to April 30, 2020, a total of 313 adult patients (≥18 years old) with laboratory-confirmed SARS-CoV-2 using PCR were enrolled. Among the 313 patients included, the mean age was 61.3 ± 14.6 years. Those who were on baseline diuretics were more likely to have hypertension (72.06% vs. 52.65% p=0.0043), congestive heart failure (25.00% vs 7.35%, p<0.001), coronary artery disease (25.00% vs 8.57%, P<0.001), chronic kidney disease (33.82% vs 11.84%, p<.001), and diabetes mellitus (55.88% vs 42.04%, p=0.042). It was also found that patients who were taking baseline diuretics also had more frequent use of ACEis and ARBs (39.71% vs 21.22% p=0.001) (Table 1).

**Table 1:** Demographic statistics by diuretic group HTN: hypertension; CVA: cerebrovascular accident; COPD: chronic obstructive pulmonary disease; CHF: congestive heart failure; CAD: coronary artery disease; CKD: chronic kidney disease; ACEi: angiotensin-converting enzyme inhibitor; ARB: angiotensin receptor blocker; BUN: blood urea nitrogen; AST: aspartate aminotransferase; ALT: alanine transaminase

Variables	Did Not Take Diuretic (n=245)	Took Diuretic (n=68)	Total (n=313)	P-value
Age, Mean (SD)	60.8 (15.2)	63.3 (11.9)	61.3 (14.6)	0.21
Male, n (%)	151 (61.63%)	37 (54.41%)	188 (60.06%)	0.28
HTN, n (%)	129 (52.65%)	49 (72.06%)	178 (56.87%)	0.0043
CVA, n (%)	14 (5.71%)	4 (5.88%)	18 (5.75%)	0.96
COPD, n (%)	20 (8.16%)	8 (11.76%)	28 (8.95%)	0.35
CHF, n (%)	18 (7.35%)	17 (25.00%)	35 (11.18%)	< .0001>
CAD, n (%)	21 (8.57%)	17 (25.00%)	38 (12.14%)	0.0002
CKD, n (%)	29 (11.84%)	23 (33.82%)	52 (16.61%)	< .0001>
Diabetes, n (%)	103 (42.04%)	38 (55.88%)	141 (45.05%)	0.042
Prior anticoagulation, n (%)	11 (4.49%)	7 (10.29%)	18 (5.75%)	0.068
Smoker, n (%)	24 (9.80%)	11 (16.18%)	35 (11.18%)	0.13
ACEi/ARB, n (%)	52 (21.22%)	27 (39.71%)	79 (25.24%)	0.0019
Cancer, n (%)	16 (6.53%)	6 (8.82%)	22 (7.03%)	0.51
Arrhythmia, n (%)	39 (15.92%)	21 (30.88%)	60 (19.17%)	0.0055
BUN				
Mean (SD)	49.0 (47.5)	92.8 (61.3)	58.5 (53.8)	< .0001>
Median (Q1, Q3)	30.0 (15.0, 70.0)	90.0 (41.0, 130.0)	37.5 (18.0, 86.0)	
Total stay in the hospital (Days)				
Mean (SD)	11.2 (8.7)	14.9 (9.5)	12.0 (9.0)	0.0044
Median (Q1, Q3)	9.0 (5.0, 15.0)	11.5 (7.0, 24.0)	9.0 (5.0, 16.0)	
Creat-Peak creatinine				
Mean (SD)	2.9 (3.4)	4.4 (3.7)	3.2 (3.5)	< .0001>
Median (Q1, Q3)	1.3 (0.9, 3.8)	2.9 (1.5, 6.7)	1.6 (0.9, 4.4)	
AST				
Mean (SD)	124.9 (289.7)	481.4 (1459.6)	201.8 (735.8)	0.0008
Median (Q1, Q3)	60.0 (34.5, 115.5)	103.5 (50.0, 195.0)	63.0 (36.0, 130.0)	
ALT				
Mean (SD)	81.5 (99.5)	237.0 (547.4)	115.0 (275.2)	0.0044
Median (Q1, Q3)	46.0 (26.0, 99.5)	82.5 (36.0, 126.0)	50.0 (27.0, 108.0)	
Outcomes				
COVID Severity, n (%)	116 (47.35%)	54 (79.41%)	170 (54.31%)	< .0001>
COVID Death, n (%)	93 (37.96%)	39 (57.35%)	132 (42.17%)	0.0042

Among 313 patients with COVID-19 who were hospitalized, there was a total of 68 (21.7%) patients taking diuretics at home and 245 (78.2%) patients who were not taking diuretics. The most used diuretics were furosemide (86%) and hydrochlorothiazide (13%). There was a total of 39 (57.35%) deaths in patients taking baseline diuretics as compared to 93 (37.96%) deaths in patients who were not taking diuretics (odds ratio {OR}: 2.2, confidence interval {CI}: (1.27 to 3.79), p=0.0047). However, after multivariable adjustment, there was no difference in overall mortality in both groups (OR:1.12; 95 % CI:0.6 to 2.1, p= 0.72) (Table 2).

**Table 2:** Association between diuretic and COVID mortality after adjusting for confounders

	Unadjusted Model		Adjusted Model	
	Odds Ratio (95% CI)	P-value	Odds Ratio (95% CI)	P-value
Took Diuretic	2.2 (1.27, 3.79)	0.0047	1.12 (0.6, 2.1)	0.72

Similar findings were noted regarding the severity of illness in COVID-19 patients. When comparing the severity of the disease, either severe or non-severe, there was a total of 54 (79.41 %) patients who had a severe illness in the diuretics group as compared to 116 (47.35%) patients who had a severe illness in the non-diuretic group, OR 4.29, CI: (2.26 to 8.13), p <.0001. After multivariable adjustment, there was no difference in overall severity in both groups. After adjusting for the confounders, there was no difference between the diuretic and non-diuretic groups (OR:1.46; CI: 0.64 to 3.3, p-value=0.36) (Table 3).

**Table 3:** Association between diuretic and COVID severity after adjusting for confounders

	Unadjusted Model		Adjusted Model	
	Odds Ratio (95% CI)	P-value	Odds Ratio (95% CI)	P-value
Took Diuretic	4.29 (2.26, 8.13)	< .0001>	1.46 (0.64, 3.3)	0.36

## Discussion

COVID-19 continues to be a global health burden causing the death of thousands of people worldwide. Like previous studies, our study showed that hypertension, diabetes mellitus, CAD, and CHF were the most common comorbidities among patients associated with COVID-19 infection. There have been several studies that have identified possible prognostic factors in COVID-19 [6]. Our research shows that among patients hospitalized with COVID-19, the baseline use of diuretics did not have a significant impact on the mortality or severity of the illness. To our best knowledge, the use of diuretics and their effect on prognosis in terms of either mortality or severity in COVID-19 has not been described.

The use of diuretics in COVID-19 patients can have both favorable and unfavorable consequences. There are limited data on the role of diuretics in COVID-19 patients, and most of the assumptions are derived from patients with acute respiratory distress syndrome (ARDS). Studies have shown that in patients with ARDS, a conservative strategy of fluid management was superior with regards to cumulative fluid balance, oxygenation index, and shortened duration of mechanical ventilation [7-8]. Recent literature advocates for the conservative fluid strategy in ARDS associated with COVID-19 patients. Loop diuretics are often used to counter the positive fluid balance in ICU patients.

Interestingly, in a recent study on 14,896 patients, Libório et al. found that loop diuretic use in critically ill patients with a positive fluid balance was associated with prolonged mechanical ventilation [9]. Similarly, another study also suggested that acetazolamide should not be used in the treatment of COVID-19, as it may carry a risk of multiple adverse consequences, including worsened ventilation-perfusion matching, impaired carbon dioxide transport, systemic hypotension, and increased work of breathing [10]. It has been postulated that with reduced cumulative fluid balance, patients are less dependent on mechanical ventilation. Although several studies describe an association between less cumulative fluid balance and reduced mechanical ventilation time [11], promoting a more negative fluid balance with diuretics is a controversial issue [12]. The time of mechanical ventilation was not studied in our study, although patients on diuretics had more length of hospitalization.

Reynolds et al. were the first to report on diuretic use with regard to the use of hydrochlorothiazide as part of a study on the effect of different classes of antihypertensive agents in COVID-19 infection. There was no significant difference in the severity of the disease [13]. Another recent study by Tsolaki V et al. suggested cautious use of diuretics in mechanically ventilated (MV) COVID-19 patients and possibly detrimental exacerbating heart-lung interactions [14]. However, this study was limited to MV patients, and the proportion of MV patients receiving diuretics was not mentioned.

Our study showed that the baseline use of diuretics in COVID-19 patients showed a tendency towards worse outcomes, even though after multivariable adjustment for confounders, there was no difference in overall mortality in both groups. There could be several reasons for this obtained result. We hypothesize that an advantage of using diuretics in trying to keep the lung "dry" is a strategy that would be helpful to avoid intubation or for successful weaning of mechanically ventilated patients. This benefit may be countered by adverse effects such as dehydration and acute kidney disease. Also, patients taking diuretics as a baseline may tend to have more comorbidities, including HTN, CHF, and CKD, than other relatively healthy patients who are taking less medication. Prior studies have shown that diuretics in critically ill patients with acute renal failure were associated with an increased risk of death [15]. However, there are no large-scale clinical studies that support the use of diuretics in COVID-19 patients.

This study had several limitations. First, this was a retrospective study conducted in New Jersey, which assessed only hospitalized patients. Therefore, these results cannot be generalized to all COVID-19 patients. Second, antiviral agents and corticosteroid usage were not included as variables in this study. Our research focused on the baseline clinical characteristics and laboratory findings related to worsening of the patients' condition due to severe disease and not on treatment. Third, this study considers only the baseline use of diuretics before admission and does not evaluate the impact of the in-patient use of diuretics or the addition of new diuretics. Finally, selection bias could not be avoided because population-based data were not used. The disease severity of patients may vary between hospitals in the same region.

## Conclusions

In conclusion, the baseline use of diuretics did not impact the overall mortality of patients infected with COVID-19. Similarly, the baseline use of diuretics did not affect the severity of the disease. Appropriate assessment of prognostic factors, baseline comorbidities, and judicious use of diuretics should be done when managing patients with COVID-19. Indeed, larger studies are needed to evaluate whether diuretic use could be an independent predictor of mortality.

## References

[1] Guan WJ, Ni ZY, Hu Y (2020). Clinical characteristics of coronavirus disease 2019 in China. N Engl J Med.

[2] Jang JG, Hur J, Choi EY, Hong KS, Lee W, Ahn JH (2020). Prognostic factors for severe coronavirus disease 2019 in Daegu, Korea. J Korean Med Sci.

[3] Vasudev R, Guragai N, Habib H (2020). The utility of bedside echocardiography in critically ill COVID-19 patients: early observational findings from three Northern New Jersey hospitals. Echocardiography.

[4] Yang G, Tan Z, Zhou L (2020). Effects of angiotensin II receptor blockers and ACE (angiotensin-converting enzyme) inhibitors on virus infection, inflammatory status, and clinical outcomes in patients with COVID-19 and hypertension. A single-center retrospective study. Hypertension.

[5] Mehra MR, Desai SS, Kuy S, Henry TD, Patel AN (2020). Cardiovascular disease, drug therapy, and mortality in Covid-19. N Engl J Med.

[6] Li X, Xu S, Yu M (2020). Risk factors for severity and mortality in adult COVID-19 inpatients in Wuhan. J Allergy Clin Immunol.

[7] Wiedemann HP, Wheeler AP, Bernard GR (2006). Comparison of two fluid-management strategies in acute lung injury. N Engl J Med.

[8] Kazory A, Ronco C, McCullough PA (2020). SARS-CoV-2 (COVID-19) and intravascular volume management strategies in the critically ill. Proc (Bayl Univ Med Cent).

[9] Libório AB, Barbosa ML, Sá VB, Leite TT (2020). Impact of loop diuretics on critically ill patients with a positive fluid balance. Anaesthesia.

[10] Luks AM, Swenson ER (2020). COVID-19 lung injury and high altitude pulmonary edema: a false equation with dangerous implications. Ann Am Thorac Soc.

[11] Mehta RL, Pascual MT, Soroko S, Chertow GM (2002). Diuretics, mortality, and nonrecovery of renal function in acute renal failure. JAMA.

[12] Grissom CK, Hirshberg EL, Dickerson JB (2015). Fluid management with a simplified conservative protocol for the acute respiratory distress syndrome*. Crit Care Med.

[13] Reynolds HR, Adhikari S, Pulgarin C (2020). Renin-angiotensin-aldosterone system inhibitors and risk of Covid-19. N Engl J Med.

[14] Tsolaki V, Zakynthinos GE, Mantzarlis K, Makris D (2020). Increased mortality among hypertensive COVID-19 patients: pay a closer look on diuretics in mechanically ventilated patients. Heart Lung.

[15] Martillo M, Garcia R, Yudelevich E, Osorio G, Shapiro J (2016). Fluid balance and diuretic administration in intubated patients undergoing weaning trials in a medical ICU. Chest.

